# Mucorales PCR for the rapid diagnosis of mucormycosis: 6 years of testing in a national reference laboratory and tertiary hospital

**DOI:** 10.1128/jcm.01911-25

**Published:** 2026-06-12

**Authors:** Allison L. Haas, Kimberly E. Hanson

**Affiliations:** 1 ARUP Laboratories 33294, Salt Lake City, Utah, USA; 2 Department of Pathology, University of Utah School of Medicine 12348 , Salt Lake City, Utah, USA; 3 Division of Infectious Diseases, Department of Medicine, University of Utah School of Medicine 12348 , Salt Lake City, Utah, USA; University of Calgary, Calgary, Alberta, Canada

**Keywords:** fungal diagnostics, fungal infection, PCR, Mucorales, mucormycosis

## Abstract

Mucormycosis is an invasive mold disease (IMD) caused by fungi in the order Mucorales. Early initiation of active antifungal therapy is critical for optimal outcomes, and molecular diagnostics can speed up the time to critical results. We reviewed 6 years of Mucorales polymerase chain reaction (PCR) testing to assess agreement with culture, histopathology, and/or pan-fungal sequencing across different specimen types. Medical record review was also performed to adjudicate cases of suspected IMD from our institution. A total of 5,779 PCRs and 2,017 fungal cultures from the same patients were performed during the study period. PCR results were issued approximately 7 days sooner than Mucorales-positive cultures. The overall PCR positivity rate was 2.9%. Sinus and other fresh tissue specimens had the highest diagnostic yield (23.8% and 19.4% PCR positive, respectively). PCR cycle thresholds (Cts) were significantly earlier for culture-positive specimens and for tissue versus other sample types, while serum had the latest Cts (all comparisons *P* < 0.005). Among 315 specimens with shared testing, PCR detected all culture- or sequencing-positive samples (*n* = 13), and negative percent agreement across methods was high (97.3%). Twenty-one cases of proven/probable mucormycosis were identified from our hospitals; 67% had at least one positive PCR. False-negative PCRs came from serum (*n* = 6) or cerebrospinal fluid (*n* = 1), while false positives were rare (PCR specificity: 99%). In conclusion, Mucorales PCR is a useful adjunct to routine testing for suspected IMD, given its relatively rapid turnaround time and high specificity. Testing should ideally be performed using fresh tissue from the primary site of infection when possible.

**IMPORTANCE:**

This work addresses a critical gap in the diagnosis of mucormycosis, a rapidly progressive and frequently fatal invasive mold disease for which timely, accurate laboratory confirmation is essential but often difficult to achieve. By evaluating over 6 years of real-world Mucorales polymerase chain reaction (PCR) testing in a national reference laboratory and tertiary-care setting, this study provides robust evidence that targeted PCR offers substantially faster turnaround times than culture while maintaining high specificity across multiple specimen types. The findings underscore the diagnostic value of molecular testing, particularly from fresh tissue obtained at the site of infection, and highlight important limitations of serum testing that can inform optimal test utilization. In the context of increasing mucormycosis incidence, absence of reliable antigen-based diagnostics, and the need for early antifungal therapy, these data support the incorporation of Mucorales PCR as a complementary tool to conventional methods.

## INTRODUCTION

Mucormycosis is a potentially life-threatening invasive mold disease (IMD) that is increasing in incidence (1, 2). The most common agents of disease include fungi in the genera *Rhizopus*, *Mucor*, and *Rhizomucor* and less commonly *Lichtheimia, Cunninghamella, Apophysomyces, Cokeromyces, Syncephalastrum, Saksenaea,* and *Actinomucor*, with predominant species varying among geographical locations. Collectively, these organisms have been identified as high-priority fungal pathogens by the World Health Organization (3).

Mucorales are commonly found in soil and decaying organic matter. These organisms are easily aerosolized, explaining their predilection to cause sinonasal, rhino-orbital-cerebral, and/or pulmonary disease (4). Invasive disease is typically associated with immunocompromising conditions such as hematologic malignancy, solid organ or hematopoietic stem cell transplantation, long-term corticosteroid use, uncontrolled diabetes, and, most recently, COVID-19 infection (5). Additionally, immunocompetent patients may be at risk for cutaneous diseases following trauma with direct inoculation of Mucorales spores.

Due to the angioinvasive nature of these molds and rapid growth rate, mucormycosis can be rapidly fatal, particularly in an immunocompromised host. In fact, Mucorales infections had the highest attributable mortality among all invasive fungal diseases (IFDs) in 2018, causing 18.6% of IFD-related deaths despite it being a relatively rare disease (1). Prompt initiation of active antifungal treatment reduces mortality (6). Thus, rapid and accurate diagnostics are essential for patient management decisions and optimal clinical outcomes. Fungal culture remains integral to the diagnosis of mucormycosis because it allows for definitive organism identification and antifungal susceptibility testing. However, culture sensitivity is relatively low (approximately 50%) and requires at least 3 days or longer to complete (7). Unlike other fungal pathogens, including *Aspergillus*, *Candida*, and *Cryptococcus*, there are currently no commercially available cell wall biomarkers for Mucorales. Molecular detection of Mucorales DNA in clinical specimens by polymerase chain reaction (PCR) is an attractive option that has the potential to improve diagnostic sensitivity as well as speed up time to results compared to culture (8, 9). In the United States, however, PCR testing options are somewhat limited. Available tests are either commercial Research Use Only reagents or are laboratory-developed tests (LDTs) and vary by the number of genera and/or species detected (10).

We developed a Mucorales PCR capable of detecting all medically important genera associated with mucormycosis in a single test. Here, we describe the results of over 6 years of Mucorales PCR testing in our national reference laboratory, evaluate positivity among various sample types, compare the turnaround time (TAT) of PCR to fungal culture and sequencing, and assess results agreement with culture, histopathology, and/or pan-fungal sequencing. Retrospective medical record reviews were also conducted to calculate test clinical performance based on adjudication of suspected IMD cases from the University of Utah (U of U).

## MATERIALS AND METHODS

**Fig 1:**
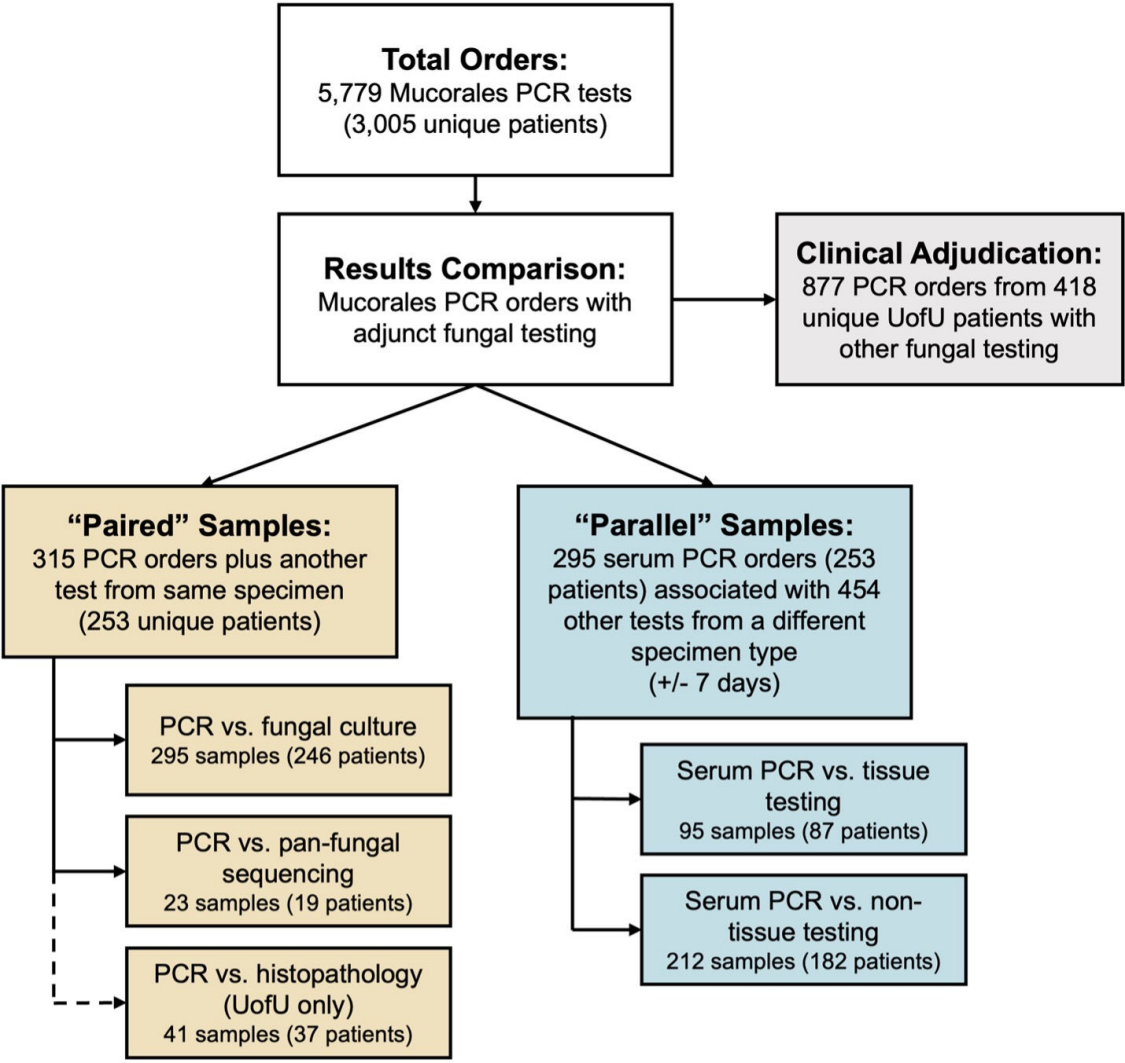
Study design. Mucorales PCR performance was evaluated by paired sample analysis, parallel sample analysis, and through clinical adjudication (for University of Utah patients only). See Materials and Methods for definitions of paired and parallel samples.

This is a retrospective observational study of Mucorales PCR testing performed as routine diagnostic testing at ARUP Laboratories (Salt Lake City, UT, USA) between July 2018 and November 2024. We compared PCR results to fungal culture, histopathology, and/or pan-fungal sequencing performed as a part of routine clinical care across a variety of different specimen types and time frames. Figure 1 illustrates test method comparisons using (i) paired specimens (i.e., different test methods performed on aliquots from the same sample) and (ii) parallel specimens (i.e., serum samples collected within 7 days of another specimen type). Test turnaround time was calculated as the time from arrival at ARUP Laboratories until result reporting and did not account for collection or transport time. We also reviewed the medical records of patients from the U of U Health System with Mucorales PCR ordered to adjudicate cases of suspected IMD in accordance with University of Utah IRB_00177982.

### Mucorales PCR assay

The ARUP Mucorales PCR assay is designed to detect, but not differentiate, the following clinically relevant Mucorales genera: *Rhizopus*, *Mucor*, *Rhizomucor*, *Cunninghamella*, *Lichtheimia*, *Apophysomyces*, *Cokeromyces*, *Syncephalastrum*, and *Saksenaea* by amplifying a 180 bp conserved region of the multi-copy 18S rRNA gene. This assay was run on the QuantStudio qPCR instrument (ThermoFisher, Waltham, MA, USA) using MGB Alert primers and probes (EliTech, Bothell, WA, USA) (Table 1). All specimens were spiked with a commercial MGB Eclipse internal control plasmid (EliTech, Bothell, WA, USA) to monitor for the presence of PCR inhibitors in patient samples (Table 1). PCR conditions included 50 cycles of amplification, with a denaturation of 95°C for 5 s, annealing at 56°C for 20 s, and extension at 76°C for 20 s. Routine quality control was monitored on each run using positive controls (culture extracts of *Mucor circinelloides*, *Rhizopus arrhizus*, *Rhizomucor* spp., or *Cunninghamella elegans*) and negative controls (base matrix).

**TABLE 1:** Primers and probes used in ARUP Mucorales PCR assay^*a*^

Probe name	Sequence (5′–3′)
Mucorales 18S	MGB-FAM-AAAT*A*CA*A*A*I*I*CCC
Internal Control	MGB-AP642-G*AATGCGGTACGTGGTCC-EDQ
Primer name	Sequence (5′–3′)
Mucorales 18S F	CA*ATCI*AAGA*AT*T*T*CACCT*CTA
Mucorales 18S R	AT*T*ACCATGAGCAAATCAGA
Internal Control F	CTGCACGGACCAGTTACTTTACG
Internal Control R	CTCATTTTTTCTACCGGAGATCTTGT

^*a*^ *Indicates modified base (EliTech).

Positive Mucorales PCR results are defined by having a cycle threshold (Ct) value of <40.0 cycles with expected amplification of the internal control. Negative Mucorales PCR results are defined as any Ct ≥40.0 cycles with expected amplification of the internal control. Specimens with Ct values >36.0 and <40.0 are repeated. Specimens with Ct values <40 on repeat are considered positive, while those with Ct values ≥40 are considered negative. All Ct values for the internal control must be within two standard deviations of the established control IC mean. Samples with IC Ct values outside of this range are repeated. If upon repeat testing, the IC Ct value is still outside of the expected range, the sample is reported as “inhibited.”

### Nucleic acid extraction

Nucleic acid extraction methods varied depending on sample type. For frozen (i.e., “fresh”) tissue, 25 mg of minced tissue was placed in lysis solution and subjected to bead beating at 6.0 m/s for 40 s. Nucleic acid was eluted in 50 μL using the Promega Maxwell RSC 48 Blood DNA and Purification Kit on the Promega Maxwell RSC 48 Instrument. Formalin-fixed paraffin-embedded (FFPE) tissues were extracted with the FFPE tissue DNA Purification Kit on the Promega Maxwell RSC 48 instrument, using three 10 μm scrolls of the tissue block eluted in 50 μL elution buffer. Body fluids, respiratory samples, and serum were extracted after bead beating using the PerkinElmer Chemagic MSM I instrument with 200 µL of raw specimen eluted in 80 μL elution buffer. For all sample types, 5 μL of the extracted DNA template was used for PCR.

### Pan-fungal sequencing

The ARUP pan-fungal identification by sequencing assay is an LDT designed to detect and identify fungi in tissue (fresh or fixed), cerebrospinal fluid (CSF), or other sterile body fluids. This test PCR amplifies the 28S rRNA and ITS2 regions of the fungal genome, followed by Sanger sequencing on the Applied Biosystems 3730 Series analyzer (ThermoFisher) with sequences analyzed using RipSeq Single (Pathogenomix). Result interpretation and performance versus histopathology have been previously described (11). Briefly, the identification of an organism to a species level requires ≥99.0% ITS2 identity with >0.8% separation between different species and ≥97.0% identity for genus-level identification. Consistent genus/species identification from both the 28S and ITS2 regions with acceptable average quality values and fewer than three ambiguous bases is also required for reporting (10).

### Fungal culture

Routine fungal culture was performed at ARUP Laboratories according to standard procedures. Briefly, primary specimens were plated on inhibitory mold, Sabouraud dextrose, and brain heart infusion agars. Per standard procedure, fresh tissue was minced and embedded in the agar. Culture plates were incubated at 30°C and monitored for growth for 35 days. Fungi isolated in culture were identified using a combination of macroscopic and microscopic characteristics, matrix-assisted laser desorption/ionization time-of-flight mass spectrometry (MALDI-ToF MS) (Bruker), and/or ITS1/ITS2 sequencing. For identification by MALDI-ToF MS, multiple databases were queried, including an in-house database, the NIH mold database, and the MSI-2 database (https://msi.happy-dev.fr).

### Adjudication of suspected IMD cases

Adult and pediatric patients from the U of U with Mucorales PCR testing documented during the study period were identified using laboratory result searches. Electronic medical records were reviewed for patients with positive mycologic results to record host risk factors for IMD, signs/symptoms of infection, 30-day all-cause mortality, adjunct laboratory results, imaging studies, histopathology, and antifungal treatment. For this cohort, suspected IMD cases were adjudicated as having “proven,” “probable,” or “possible” invasive mucormycosis by applying the European Organization for Research and Treatment of Cancer/Invasive Fungal Infections Cooperative Group and the National Institute of Allergy and Infectious Diseases Mycoses Study Group (EORTC/MSG) updated consensus definitions (12). In addition, we modified these definitions to also apply to patients with additional risk factors for mucormycosis other than hematologic malignancy and/or transplantation (Table S1). We did not include Mucorales PCR as mycologic evidence of disease. However, we presumed that patients with negative Mucorales PCRs, negative fungal cultures, and/or pan-fungal sequencing were either “possible” or unlikely cases of mucormycosis for the sake of clinical adjudication.

### Statistics

All statistical analyses were performed in GraphPad Prism version 10.0 (San Diego, CA, USA). Data figures are presented as the median and interquartile range, as shown in the figure legend. Where indicated, a two-tailed Student’s *t* test or one-way ANOVA followed by Dunnett’s *post hoc* test was used to determine statistical significance. Statistical significance was defined as *P* < 0.05. The sensitivity and specificity of Mucorales PCR were determined using the composite mucormycosis case definition as the reference standard.

## RESULTS

### PCR results

**Fig 2:**
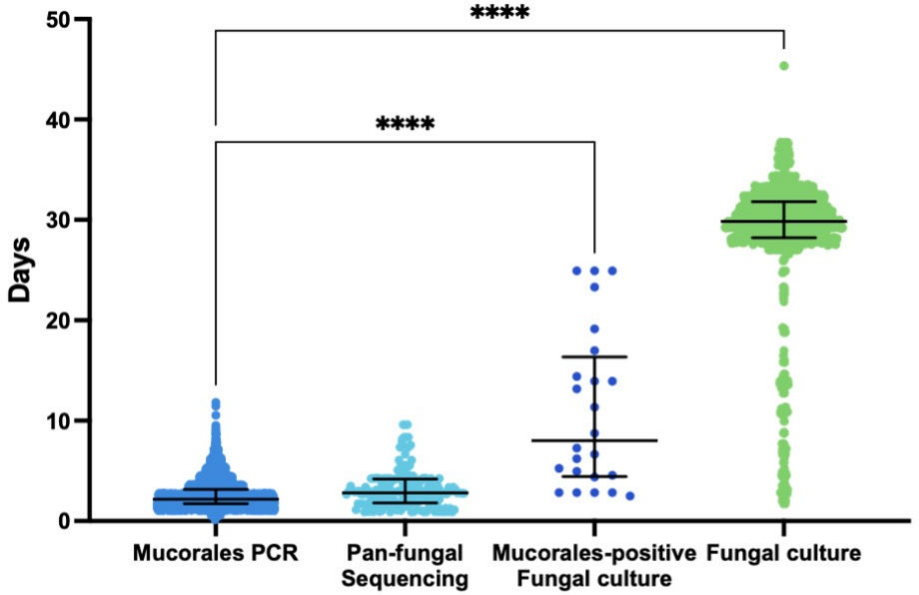
Fungal testing turnaround time. Total time from arrival at the laboratory to final result reporting for all samples analyzed for this study. Median and interquartile range are plotted (black bars). Mucorales PCR (*n* = 5,779), pan-fungal sequencing (*n* = 198), Mucorales-positive fungal culture (*n* = 24), and fungal culture (*n* = 2,917). ****P < 0.0001.

During the 6-year study period, 5,779 Mucorales PCR tests from 3,005 patients were performed (Fig. 1). Most testing came from institutions other than the U of U (84.8%). The average age of patients was 57 years (range 5 days–93 years), and most were male (61.1%). In total, 165 samples were positive for Mucorales DNA (2.9% positivity rate), and 20 were inhibited. The median turnaround time for PCR result was 2.4 days (range 0.1–11.8 days) versus 9.3 days (range 2.5–24.9 days) for Mucorales-positive cultures (difference of 6.9 days faster) (Fig. 2).

PCR testing was most commonly ordered on bronchoalveolar lavage (BAL) (66.0%) and serum (23.4%) samples, but positivity rates varied significantly across specimen types (Table 2). Tissue and abscess fluid had the highest positivity (17.6% and 25%, respectively), while CSF and sputum had the lowest (0% and 1.4%, respectively). Of all tissue sources, sinus and “nasal” tissues had the highest positivity rates (23.8%). Additionally, we observed a higher positivity rate among fresh (frozen) tissues (19.4%) compared to fixed tissues (13.3%).

**TABLE 2:** Mucorales PCR positivity by specimen type^*a*^

Specimen type	Total	Positive	% Positivity
Respiratory (all)	3,921	94	2.4
BAL	3,817	93	2.4
Sputum	71	1	1.4
Serum	1,351	30	2.2
Body fluid (all)	65	8	12.3
Pleural fluid	47	6	12.8
Tissue (all)	153	27	17.6
Lung	43	6	14.0
Sinus/nasal	31	5	23.8
FFPE/fixed	30	4	13.3
Fresh	62	12	19.4
Abscess fluid	4	1	25.0
Other	133	1	0.8
CSF	29	0	0
Source not provided	151	4	2.6
Total	5,779	165	2.9

^*a*^ Total number, number positive, and percent positivity of specimens tested at ARUP Laboratories stratified by source. BAL, bronchoalveolar lavage; FFPE, formalin-fixed paraffin-embedded tissue; and CSF, cerebrospinal fluid.

**Fig 3:**
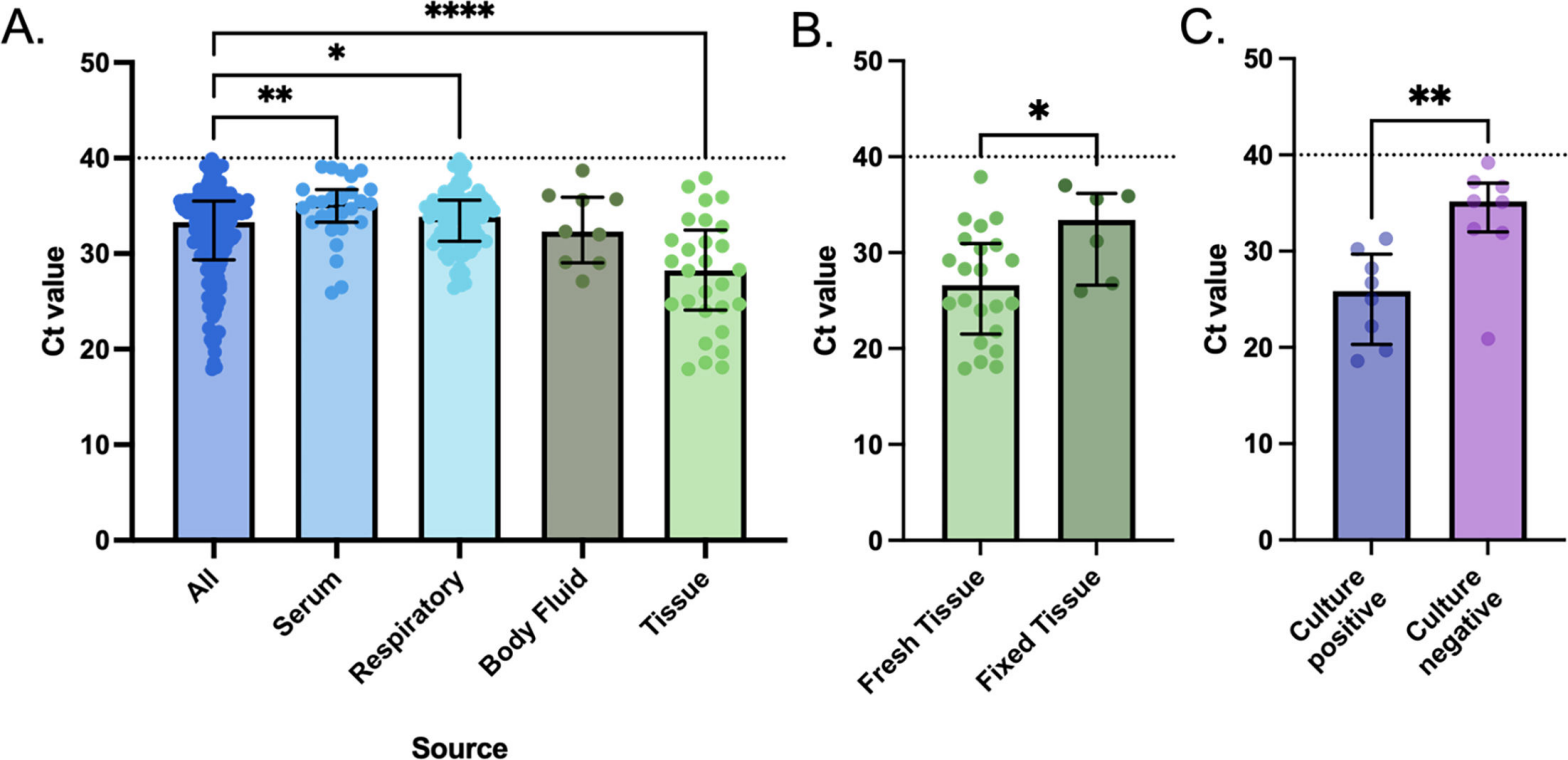
Cycle threshold values by specimen type. (**A**) Ct values from all positive Mucorales PCR results categorized by specimen type. (**B**) Ct value of tissue specimens separated by fresh (i.e., non-fixed) versus fixed tissue samples. (**C**) Ct values from PCR-positive samples (any source) with a paired fungal culture, separated by culture positivity. Median and interquartile range are shown (black bars). Any Ct ≥40.0 was considered negative (dashed line). *P < 0.05, **P < 0.01, and ****P < 0.0001.

Ct values were compared across specimen types and by culture results (Fig. 3A through C). Tissue specimens had significantly lower Ct values overall (median 28.3, IQR 24.1–32.5) compared with all other specimen types, signifying a higher organism burden, whereas serum samples had significantly higher Ct values (35.3, IQR 33.3–36.7), suggestive of low levels of circulating DNA. Additionally, fresh tissues had significantly lower Ct values compared to fixed tissues (26.6, IQR 21.5–31.0 and 33.4, IQR 26.6–36.2, respectively) (Fig. 3B). PCR results with positive paired fungal cultures also had significantly lower Ct values suggestive of higher organism burden, with culture-positive specimens often having Ct values of ≤31 cycles versus >32 from culture-negative (non-serum) specimens (Fig. 3C; Table 3).

**TABLE 3:** Paired samples with at least one positive fungal result^*a*^

Patient number	Sample type	Mucorales PCR Ct value	Culture	Pan-fungal sequencing	Mucormycosis case definition
9	Tissue	18.6	*Rhizopus arrhizus*	*Rhizopus* spp.	Proven
4	Tissue	19.7	*Mucor circinelloides*	*Mucor* spp.	Proven
11	BAL	20.9	*Rhizopus* spp.	n/p	Probable
1	Tissue	22.2	*Rhizopus* spp.	*Rhizopus* spp.	Proven
10	Sinus tissue	25.0	*Rhizopus* spp. *Aspergillus flavus*	*Rhizopus* spp.	Proven
6	BAL	26.7	*Rhizopus* spp.	n/p	Proven
3	Sinus tissue	28.2	*Rhizopus* spp.	*Rhizopus* spp.	Proven
6	BAL	30.2	*Rhizopus* spp.	n/p	Proven
12	BAL	31.3	*Rhizopus* spp.	n/p	Proven
5	BAL	31.9	Negative	n/p	Possible
12	BAL	32.3	Negative	n/p	Proven
13	BAL	35.1	Negative	n/p	Possible
2	Serum	35.2	Negative	n/p	Possible
N/a^*b*^	BAL	36.7	Negative	n/p	N/a^*b*^
8	BAL	37.2	Negative	n/p	Possible
7	BAL	39.2	Negative	n/p	Possible

^*a*^ Positive mucorales PCR samples shared with fungal culture and/or pan-fungal sequencing were clinically adjudicated according to the modified EORTC/MSG criteria for mucormycosis, as presented in Table S1. n/p, not performed; N/a, not available.

^*b*^ Indicates patient from outside the U of U.

### Paired sample comparisons

A total of 315 samples from 253 patients had at least two fungal test methods, including Mucorales PCR performed simultaneously on the same sample. Overall, 295 paired samples had both PCR and culture performed, while 23 had both PCR and sequencing completed. Of the paired samples, 16 (5.1%) were positive by Mucorales PCR, 9 had Mucorales recovered in culture (3.1%), and 5 had Mucorales DNA detected by pan-fungal sequencing (2.7%) (Table 3). All culture and/or pan-fungal sequencing Mucorales-positive specimens were also PCR positive. Overall negative percent agreement across methods was 97.3% (291/299), with a negative percent agreement of 97.2% (279/289) and 100% (18/18) compared to fungal culture and pan-fungal sequencing, respectively. Importantly, 64 of the paired specimens had potential fungal pathogens other than Mucorales recovered in culture (Table S2). All of these were negative by PCR, suggesting no cross-reactivity with other common fungi. Tissue PCR agreement with histopathology was also assessed for the U of U patients only (Table S3). Of 39 patients with paired PCR and histopathology, 5 had a positive Mucorales PCR result, and all had fungal elements observed in tissues. Of these, four were described as consistent with mucormycosis by the pathologist, and one had generic “fungal elements” reported. One of the PCR-positive/tissue histopathology compatible with Mucorales specimens was negative by pan-fungal sequencing. There were no patients who had fungal elements consistent with mucormycosis that had negative Mucorales PCR results, resulting in a 100% agreement with paired tissue histopathology.

### Parallel sample comparisons

A total of 253 patients had Mucorales serum PCR testing performed in parallel (±7 days) with culture and/or pan-fungal sequencing; 87 patients had parallel serum testing with tissue samples, and 182 patients had parallel serum testing with non-tissue specimens, including respiratory, body fluid, and/or CSF specimens. Of the patients with parallel tissue testing, 14 had Mucorales detected by at least one method (Table 4). Five of six serum PCR-positive patients also had positive tissue results, while eight tissue-positive patients had negative serum PCRs (i.e., false-negative serum PCRs). Of note, one of the false-negative serum PCRs had an amplification curve detected, but greater than the cutoff used to define a positive test (Patient 16 [45.5], Table S4). Of the patients with parallel non-tissue testing, three were serum PCR positive, but only one also had Mucorales spp. detected by culture/sequencing in a non-tissue specimen (BAL). No false-negative serum PCRs were observed in the non-tissue subgroup (Table S4).

**TABLE 4:** Serum Mucorales PCR with parallel tissue samples^*a*^

Patient number	Serum PCR result (Ct)^*b*^	Tissue PCR result (Ct)	Tissue fungal culture	Tissue pan-fungal sequencing
6	+ (34.2)	n/p	*Rhizopus* spp.	n/p
2	+ (35.2)	n/p	Negative	n/p
23	+ (36.7)	n/p	Negative	*Rhizopus* spp.
15	+ (39.0), − (50.0)	n/p	*Rhizopus arrhizus*	n/p
25	+ (39.1)	n/p	*Rhizopus arrhizus*	n/p
27	+ (39.4)	n/p	n/p	*Cunninghamella/ Rhizopus* spp.
16	− (45.5)	n/p	*Rhizopus* spp.	*Rhizopus* spp.
1	− (50)	+ (22.2)	*Rhizopus* spp.	*Rhizopus* spp.
3	− (50)	+ (28.2)	*Rhizopus* spp.	*Rhizopus* spp.
14	− (50)	n/p	*Rhizopus* spp.	n/p
18	− (50)	n/p	*Rhizopus arrhizus*	n/p
21	− (50)	n/p	*Rhizopus arrhizus*	Negative
26	− (50)	n/p	*Rhizopus arrhizus*	*Rhizopus* spp.
N/a^*c*^	− (50)	n/p	n/p	*Rhizopus* spp.

^*a*^ Patients with positive serum mucorales PCR or other positive mucorales results from tissue samples (i.e., culture and/or pan-fungal sequencing) with corresponding test results performed within 7 days of each other. n/p, not performed; N/a, not available.

^*b*^ Positive < 40.0.

^*c*^ Patient from outside the U of U.

### Clinical performance

Of 418 U of U patients tested, 27 had a positive Mucorales result; of these, 77.8% (21/27) were classified as “proven” or “probable” cases of mucormycosis using the modified EORTC/MSG criteria for mucormycosis (Tables S1 and S5). The overall disease prevalence (proven/probable infection) was 5.0% among patients with suspected mucormycosis as defined by ordering a dedicated Mucorales PCR (Table 5). There were four pulmonary, seven sinus, six skin/soft tissue, three rhino-orbito-cerebral, and one disseminated infections identified during the study period. Of the 21 proven/probable cases, 14 had at least one positive PCR result, demonstrating an overall PCR sensitivity (agnostic of specimen type) of 66.7% (95% CI: 43.0%–85.4%), specificity of 98.5% (96.7%–99.4%), negative predictive value of 98.2% (96.8%–99.0%), and positive predictive value of 70.0% (49.9%–84.5%) (Table 5).

**TABLE 5:** Clinical test performance^*a*^

Clinical agreement		
	Mucorales PCR positive	Mucorales PCR negative
Proven/Probable mucormycosis	14	7
Possible/No mucormycosis	6	391
**Test Performance**		
	**Value**	**95% CI**
Sensitivity	66.67%	43.03% to 85.41%
Specificity	98.49%	96.74% to 99.44%
Positive Likelihood Ratio	44.11	18.86 to 103.18
Negative Likelihood Ratio	0.34	0.18 to 0.62
Disease Prevalence	5.02%	3.14% to 7.58%
Positive Predictive Value	70.00%	49.94% to 84.51%
Negative Predictive Value	98.24%	96.83% to 99.03%
Accuracy	96.89%	94.74% to 98.33%

^*a*^ Mucorales PCR clinical test performance prior to discrepant resolution for all sample types. Refer to Table S5 for adjudication of proven, probable, and possible mucormycosis.

False-negative PCRs mostly came from serum (5/6) and one CSF; all were cases of isolated cutaneous or sinus disease (Table S6). Interestingly, three false-negative PCRs (two serum and one CSF) had amplification curves with Ct values greater than our cutoff established to define a positive test (range 45.5–48.2 cycles). In addition, the Mucorales PCR was positive in six patients with no other mycologic criteria for mucormycosis; three had proven IMD based on histopathology and, therefore, were likely true positive PCRs, while the others met the EORTC/MSG case definition for “possible” IMD (Table S6). Of the three possible IMD cases, two were treated for suspected mucormycosis, while one was never treated and improved. The latter patient was deemed to have a potentially false-positive PCR result (BAL, Ct 35.1). Following final case assessment, the Mucorales PCR assay demonstrated overall sensitivity of 73.1% (95% CI: 52.2%–88.4%), specificity of 99.7% (98.6%–99.9%), negative predictive value of 98.2% (96.7%–99.1%), and positive predictive value of 95.0% (72.6%–99.3%) (Tables 5 and 6).

**TABLE 6:** Clinical test performance following final case assessment^*a*^

Clinical agreement		
	Mucorales PCR positive	Mucorales PCR negative
Proven/Probable mucormycosis	19	7
Possible/No mucormycosis	1	391
**Test Performance**		
	**Value**	**95% CI**
Sensitivity	73.08%	52.21% to 88.43%
Specificity	99.74%	98.59% to 99.99%
Positive Likelihood Ratio	286.46	39.89 to 2056.91
Negative Likelihood Ratio	0.27	0.14 to 0.51
Disease Prevalence	6.22%	4.10% to 8.98%
Positive Predictive Value	95.00%	72.57% to 99.27%
Negative Predictive Value	98.24%	96.74% to 99.06%
Accuracy	98.09%	96.26% to 99.17%

^*a*^ Refer to Table S6 for discrepant resolution and final case assessment details.

## DISCUSSION

Mucormycosis is a difficult-to-diagnose, potentially life-threatening infection caused by fungi in the order Mucorales. The clinical, radiographic, and histopathologic signs of mucormycosis are not organism-specific, but differentiating Mucorales infection from other IMDs is essential for guiding optimal antifungal therapy. Conventional diagnostic tests for mucormycosis include direct specimen examination, culture, and histopathology. Culture remains the microbiologic reference standard but misses approximately 50% of invasive Mucorales infections (13) and requires 3 days or more to observe growth (14). Currently, there are no rapid antigen tests for Mucorales, although these are under development (15). Instead, recent diagnostic advances for mucormycosis have largely come from the development and validation of real-time PCR-based tests. Mucorales PCRs targeting the fungal 18S, 28S, and/or the ITS rDNA regions are well described in the literature (10) and have been shown to have low interlaboratory variability in Ct values when testing a standardized set of serum specimens despite 26 different protocols being used for testing (16). Whether the same agreement holds true for other specimen types, however, has not been assessed.

Our large real-world study adds to the growing evidence in support of Mucorales PCR diagnostic utility. PCR test turnaround time was significantly faster than culture (almost 9 days on average), which has potentially important implications for prompt therapeutic decision-making. An important caveat here is that we did not account for the shipping time required to send testing out to our reference laboratory, and not all specimens sent to our laboratory had PCR and culture ordered concurrently. The clinical benefits of a more rapid TAT are likely best realized when testing is performed nearer to the patient when feasible.

Our Mucorales PCR positivity rate was low overall (2.9%), but the test volume was relatively high, which is in line with the fact that mucormycosis is a dreaded, yet rare disease with non-specific clinical manifestations. Fresh tissue, including sinus specimens, had the highest positivity rates, reinforcing the benefits of tissue testing from the site of suspected infection when possible. Higher positivity rates from tissue relative to other direct testing (e.g., from BAL or other sterile body fluids) may have been influenced by a higher pre-test probability, especially if histopathology or frozen sections performed locally revealed fungal elements, and this influenced the decision to send PCR testing to our laboratory. Additionally, fresh tissue had lower Ct values than fixed tissues, likely because formalin fixation degrades DNA, and PCR inhibitors may be present in these samples. The reduced sensitivity of fixed tissue compared to fresh tissue has also been previously described for other Mucorales PCR platforms (8). For these reasons, testing from fresh tissues may be preferred over fixed specimens when there is a high clinical suspicion for IMD.

Despite access to a large amount of test results, clinical test performance could only be determined for patients from the U of U, for whom we had access to medical records. In the clinical case review, Mucorales PCR had good overall sensitivity (73.1%) with excellent specificity (99.7%) versus a composite case definition of mucormycosis. After clinical case review, five of six PCR-positive-only cases were felt to be consistent with Mucorales infection and were treated as such by the clinical team caring for the patient. Due to the small number of U of U patients with proven/probable mucormycosis, we were unable to calculate test performance by specimen type. Additionally, given the low prevalence of disease in our cohort, the negative predictive value of PCR was excellent (98.2%). False-negative PCRs generally came from serum, likely because circulating DNA was either absent in localized infections or was below our analytically validated test cutoff. Of the U of U patients with proven/probable mucormycosis, 11 had serum PCR testing ordered in parallel with other specimens. Of these, five patients had positive serum PCR results, and six patients had negative serum PCR results (Tables S1 and S5). Notably, half of the false-negative serum PCR results came from patients with localized cutaneous disease, suggesting that levels of circulating DNA may be dependent on the severity and extent of disease. Additionally, future adjustment of our assay positivity threshold could be considered to account for sample types with potential for lower levels of circulating DNA (e.g., serum and CSF).

Despite potential for lower sensitivity, as observed in this study and others (8), the benefits of serum testing are the non-invasive nature of specimen collection, potential for earlier detection of infection, and the prognostic value of repeated positive results, both in terms of attributable mortality and treatment response (9). Similar advantages apply to plasma testing (17, 18). Our assay was validated for serum testing only. Whether serum performs differently from plasma has not been assessed for Mucorales and should be a focus of future studies. Additionally, the impact of PCR amplicon size on the detection of circulating Mucorales DNA in blood samples has not been directly assessed. Circulating microbial cell-free DNA (mcfDNA) is often present in small fragments, shorter than human-derived cfDNA (19). The amplicon size of our Mucorales PCR is 180 bp, slightly larger than mcfDNA-specific assays (17). However, the exact size of circulating Mucorales DNA, and whether this DNA is truly cell-free, present in extracellular vesicles, or is organism-associated due to angioinvasion, has not been established. Recently, larger serum sample volumes for nucleic acid extraction (i.e., 1 mL) and DNA template volumes (≥7 µL) for PCR amplification were shown to significantly improve the performance of serum Mucorales PCR (20). These volume adjustments could also be modifications to enhance the analytic sensitivity of our assay in the future.

For patients with suspected IMD, deciding whether to order a targeted fungal PCR, pan-fungal sequencing, or both in addition to culture can be difficult when molecular tests are available. We have not directly compared the analytical sensitivity of our Mucorale*s* PCR and pan-fungal sequencing assay. However, the Mucorales PCR assay involves more amplification cycles than our sequencing assay, theoretically enhancing sensitivity. In this study, pan-fungal sequencing missed a single PCR-positive specimen, but the number of paired specimens with both tests performed simultaneously was small. There were cases, however, where pan-fungal sequencing (and/or culture) identified pathogenic fungi other than Mucorales. Furthermore, the pan-Mucorales PCR design does not differentiate members of the order. While the benefit of a pan-Mucorales assay is its inclusivity for many clinically relevant genera, specific identification of certain genera may be desirable due to variable antifungal susceptibility patterns and concerns for antifungal resistance (21, 22).

**Fig 4:**
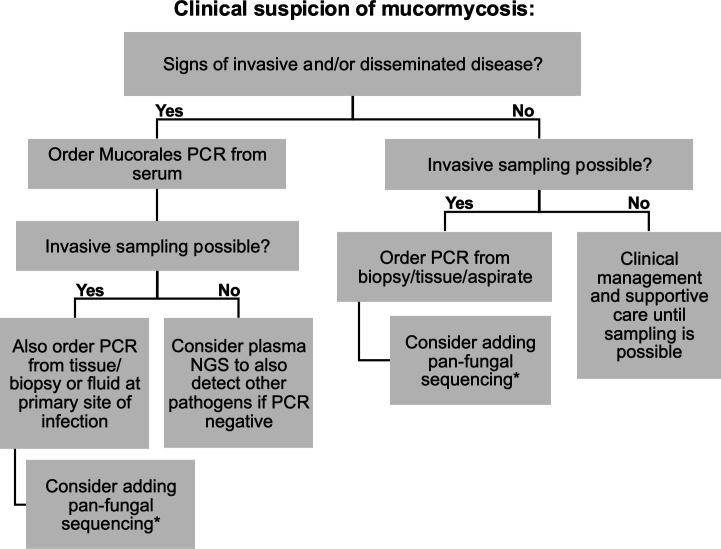
Potential Mucorales PCR ordering algorithm. For patients with appropriate host risk factors and clinical suspicion of mold infection, careful selection of specimen type for molecular testing is required for optimal diagnostic yield. *Pan-fungal Sanger sequencing is only recommended for normally sterile samples. Next-generation sequencing has the potential benefit of resolving mixtures of fungi in non-sterile samples.

With a clinical picture consistent with mucormycosis and fungal elements matching Mucorales observed in tissue, performing targeted PCR first is reasonable. If the PCR is negative, pan-fungal sequencing could then be performed as a follow-up test. However, because IMD clinical signs and symptoms are non-specific and the appearance of fungal hyphae in tissue is often neither straightforward nor organism-specific, sequence-based identification may be preferred over targeted PCR when non-descript fungal elements are seen and/or when additional fungi are in the differential diagnosis. Figure 4 illustrates a potential scheme for fungal molecular diagnostic test utilization, recognizing that this will vary by institution and with the availability of testing. Additional targeted PCRs for other agents of IMD may also be considered in conjunction with Mucorales PCR due to similarities in clinical presentation and risk factors.

In conclusion, Mucorales PCR is an accurate and relatively rapid test that provides diagnostic value for patients with suspected IMD using a variety of different specimen types. The test, however, does not replace culture, which is required to obtain an isolate for definitive organism identification, susceptibility testing, and to potentially detect infections with mixtures of fungi, which are not possible using targeted PCR or Sanger sequencing. Targeted Mucorales PCR may be preferred over pan-fungal sequencing when compatible fungal elements are seen in tissue, and the clinical suspicion for mucormycosis is high due to lower cost, potential for more rapid turnaround, and potential for higher sensitivity. With increased availability and adoption of local nucleic acid amplification testing across the world, the advantages of Mucorales PCR testing will continue to become better realized.

## SUPPLEMENTAL MATERIAL

Tables S1 to S6 jcm.01911-25-s0001.docx Modified definitions for mucormycosis and summaries of all microbiologic and histopathologic results.

ASM does not own the copyrights to Supplemental Material that may be linked to, or accessed through, an article. The authors have granted ASM a non-exclusive, world-wide license to publish the Supplemental Material files. Please contact the corresponding author directly for reuse.
