# Supplementary material for: Mucorales PCR for the rapid diagnosis of mucormycosis: 6 years of testing in a national reference laboratory and tertiary hospital
Source: J Clin Microbiol. 2026 Jun 12;64(7):e01911-25. doi: 10.1128/jcm.01911-25 (PMC13343893; doi:10.1128/jcm.01911-25)
Supplement: Tables S1 to S6 — Modified definitions for mucormycosis and summaries of all microbiologic and histopathologic results. [file jcm.01911-25-s0001.docx]

**Supplemental Table 1. Modified EORTC/MSG Criteria for Mucormycosis**

| **2020 criteria for Invasive Mold Disease**  **(IMD)** (13) | **Modified definitions for mucormycosis** |
| --- | --- |
| **Proven*** | |
| Histopathologic, cytopathologic, or direct microscopic evidence of hyphae and evidence of tissue damage from needle aspiration or biopsy | Observation of fungal elements and Mucorales spp. DNA detected by sequencing or Mucorales spp. recovered in culture from same site. |
| Recovery of mold in culture from a specimen collected using sterile technique from a normally sterile site and clinical or radiologic abnormalities consistent with infectious process | Recovery of Mucorales spp. in culture from a specimen obtained from a sterile procedure from a normally sterile site with disease consistent with mucormycosis |
| Amplification of fungal DNA by PCR combined with DNA sequencing when molds are observed in FFPE tissue |  |
| **Probable**** | |
| Appropriate host factors: Neutropenia (>10 days), hematologic malignancy, Allogeneic stem cell transplant, solid organ transplant, prolonged corticosteroid use, use of T- or B-cell immunosuppressants, inherited severe immunodeficiency, acute graft-versus-host disease refractory to steroid treatment | All IMD host factors, including uncontrolled diabetes, trauma and/or recent COVID-19 |
| Clinical and radiologic evidence (see Donnelly *et al.,* CID (2020) for breakdown depending on disease state) | Clinical and radiologic evidence including tissue necrosis (cutaneous mucormycosis), pulmonary findings (including consolidation, lesions, nodules, cavitations), or sinusitis (including mucosal thickening, bony erosion, opacification) |
| Mycological evidence: Mold recovered in culture, microscopic detection of fungal elements from sputum, BAL, bronchial brush, or aspirate | Recovery of Mucorales spp. in culture from any other sources |
| **Possible** | |
| Appropriate host factor (see above) | (as above) |
| Clinical evidence consistent with IFD | (as above) |

*Can apply to any patient regardless of immune status

******Probable invasive mold disease (IMD) requires the presence of at least 1 host factor, a clinical feature, and mycologic evidence and applies to immunocompromised patients only

**Supplemental Table 2.** Other fungi recovered in culture in samples shared with Mucorales PCR*

| **Number of specimens** | **Organism recovered in culture** |  | **PCR result** |
| --- | --- | --- | --- |
| 2 | *Aspergillus fumigatus* |  | Negative |
| 1 | *Aspergillus terreus* group |  | Negative |
| 4 | *Candida albicans* |  | Negative |
| 1 | *Coccidioides immitis* |  | Negative |
| 1 | *Fusarium spp.* |  | Negative |
| 1 | *Histoplasma capsulatum* |  | Negative |
| 1 | *Nakaseomyces (Candida) glabratus* |  | Negative |
| 3 | *Penicillium spp.* |  | Negative |
| 3 | *Saccharomyces cerevisiae* |  | Negative |
| 1 | *Scedosporium apiospermum* |  | Negative |
| 46 | Yeast, not otherwise specified |  | Negative |
|  | | | |
| Total = 64 | | | |

*From any specimen source

**Supplemental Table 3. University of Utah tissue specimens with paired* testing**

| **Patient Number** | **Source** | **Tissue type** | **Mucorales PCR result (Ct)** | **Fungal elements present? (and description)** | **Pan-fungal sequencing performed?** | **Diagnosis** |
| --- | --- | --- | --- | --- | --- | --- |
| 339 | Lung | Fresh | Not Detected | No | no | No infectious agent identified |
| 271 | Forearm | Fresh | Not Detected | No | no | No infectious agent identified |
| 36 | Nasal | Fresh | Not Detected | No | no | No infectious agent identified |
| 413 | Lung | Fresh | Not Detected | No | Yes; negative | No infectious agent identified |
| 374 | Lung | Fresh | Not Detected | No | Yes; negative | No infectious agent identified |
| 216 | Lung | Fresh | Not Detected | Yes; "presence of fungal hyphae" | Yes; negative | No infectious agent identified |
| 115 | Abdomen | Fresh | Not Detected | Yes; "Fungal spores" | Yes; *Candida albicans* | *Candida albicans* |
| 337 | Lung | Fresh | Not Detected | No | no | No infectious agent identified |
| 111 | Lung | Fresh | Not Detected | Yes; "Small yeast forms. Occasional larger fungal elements resembling possible spherules or possible fragments of fruiting bodies… possibility of *Coccidioides* or *Aspergillus*." | no | *Coccidioides immitis* |
| 3 | Orbit | Fresh | Detected (28.2) | Yes; "Presence of fungal elements" | Yes; *Rhizopus* spp. | *Rhizopus spp.* |
| 542 | Lung | Fresh | Not Detected | Yes; "Fungal hyphal elements appear to show irregular branching with some acute angle branching and possible septation." | no | *Aspergillus fumigatus* |
| 319 | Lung | Fresh | Not Detected | No | no | No infectious agent identified |
| 462 | Lung | Fresh | Not Detected | No | no | No infectious agent identified |
| 338 | Lung | Fresh | Not Detected | No | no | No infectious agent identified |
| 142 | Lung | Fresh | Not Detected | No | Yes; negative | *Nocardia paucivorans & Candida dubliniensis* |
| 152 | Nares | Fresh | Not Detected | No | no | No infectious agent identified |
| 338 | Lung | Fresh | Not Detected | No | no | No infectious agent identified |
| 9 | Nose | Fresh | Detected (18.6) | Yes; "Invasive fungal elements, morphologically compatible with mucormycosis" | Yes; *Rhizopus spp.* | *Rhizopus arrhizus* (formerly *R. oryzae*) |
| 1 | Buttock | Fresh | Detected (22.2) | Yes; "Morphologically consistent with mucormycosis" | Yes; *Rhizopus spp.* | *Rhizopus arrhizus* (formerly *R. oryzae*) |
| 95 | Lung | Fresh | Not Detected | No | no | No infectious agent identified |
| 371 | Lung | Fresh | Not Detected | No | no | No infectious agent identified |
| 53 | Lung | Fresh | Not Detected | No | no | No infectious agent identified |
| 72 | Lymph node | Fresh | Not Detected | No | Yes; negative | No infectious agent identified |
| 207 | Lung | Fresh | Not Detected | No | no | No infectious agent identified |
| 381 | Lung | Fresh | Not Detected | No | Yes; negative | No infectious agent identified |
| 218 | Lung | Fresh | Not Detected | No | no | No infectious agent identified |
| 161 | Sinus | Fresh | Not Detected | Yes; "Positive for fungal elements, involving vessels" | no | *Fusarium spp.* |
| 255 | Lung | Fresh | Not Detected | No | Yes; negative | Influenza A |
| 270 | Nasal | Fresh | Not Detected | Yes; "Invasive fungal sinusitis with angioinvasion" | Yes; *Fusarium spp.* | *Fusarium spp.* |
| 10 | Sinus | Fresh | Detected (25.0) | Yes; "Invasive fungal hyphal elements showing a broad ribbon-like appearance and no septations are compatible with Mucorales species." | Yes; *Rhizopus spp.* | *Rhizopus spp.*& *Aspergillus flavus* |
| 445 | Soft palate | Fresh | Not Detected | Yes; "Fungal elements suggestive of *Candida* species" | no | No infectious agent identified |
| 8 | Lung | Fresh | Not Detected | Yes; "Fungal elements with thin, branching, septate hyphae, consistent with *Aspergillus*." | Yes; *Aspergillus spp.* | *Aspergillus* |
| 158 | Scalp | Fresh | Not Detected | Yes; "Hyphae are septated, measure between 5 and 10 µm in diameter and exhibit acute to 90 degree branching." | Yes; *Scedosporium spp.* | *Scedosporium apiospermum* |
| 350 | Ankle | Fresh | Not Detected | No | Yes; negative | No infectious agent identified |
| 463 | Lung | Fresh | Not Detected | No | Yes; negative | No infectious agent identified |
| 20 | Eustachian tube | Fixed | Detected (26.8) | Yes; "Fungal organisms morphologically compatible with Zygomycetes spp. (Mucor)" | Yes; negative | Mucorales (only detected by PCR) |
| 270 | Nasal | Fixed | Not Detected | Yes; "Invasive fungal sinusitis with angioinvasion" | Yes; positive for *Fusarium spp.* | *Fusarium spp.* |
| 7 | Brain | Fixed | Not Detected | No | no | *Toxoplasma* |
| 260 | Lung | Fixed | Not Detected | No | Yes; negative | No infectious agent identified |

*Defined as testing using the same specimen from the same procedure identified by specimen type and date/time of collection

**Supplemental Table 4. Patients with parallel serum *Mucorales* PCR**

| **Pt #** | **Serum PCR (Ct)** | **Other Mucorales PCR (Ct) (source)** | **Risk factors** | **Clinical Disease** | **Dissemination**** | **Angioinvasion***** | **Fungal Culture** | **Pan-fungal Sequencing** |
| --- | --- | --- | --- | --- | --- | --- | --- | --- |
| 6 | **+ (34.2)** | + (26.7) (BAL) + (30.2) (BAL) | SOT | Disseminated | Yes | Yes | *Rhizopus spp.* (BAL) *Rhizopus spp.* (Pericardial fluid)  *Rhizopus spp.* (Lung tissue)  *Rhizopus spp.* (Pericardial tissue) | n/p |
| 2 | **+ (35.2)** | n/p | HDCS | Sinusitis | Yes | No | *Aspergillus fumigatus* (Lung tissue) *Candida tropicalis* (Blood) | n/p |
| 17 | **+ (35.8), + (38.7)** | - (50) (BAL) | HSCT, NP | Pulmonary | No | n/p | Negative (BAL) | n/p |
| 23 | **+ (36.7)** | n/p | HM | Cutaneous | No | No | Negative (Skin tissue) | *Rhizopus spp.* |
| 15 | **+ (39.0), - (**50.0) | n/p | HM | Sinusitis | No | No | *Rhizopus arrhizus* (Sinus tissue) | n/p |
| 25 | **+ (39.1)** | n/p | UDM | Cutaneous, Disseminated | Yes | Yes | *Lichtheimia corymbifera* (Sinus tissue) | n/p |
| 27 | **+ (39.4)** | - (50) (BAL) | UDM | Pulmonary | No | No | *Nakaseomyces glabratus* (BAL) *Nakaseomyces glabratus* (sputum) | *Cunninghamella/ Rhizopus spp.* |
| 16 | - (45.5) | n/p | SOT | Cutaneous | No | Yes | *Rhizopus spp.* (Leg tissue) *Rhizopus spp.* (Leg tissue) | *Rhizopus spp.* |
| 1 | - (50) | + (22.2) (Skin tissue) | HM | Cutaneous | No | No | *Rhizopus spp.* (Sinus tissue) Negative (Blood) | *Rhizopus spp.* |
| 3 | - (50) | + (28.2) (Sinus tissue) | HSCT | Sinusitis | No | No | *Rhizopus spp.* (Sinus tissue) | *Rhizopus spp.* |
| 14 | - (50) | n/p | HDCS | Sinusitis | No | Yes | *Rhizopus spp.*  (Sinus tissue) | n/p |
| 18 | - (50) | - (50) (BAL) | none | Cutaneous | No | Yes | *Lichtheimia corymbifera* (Leg tissue) Negative (BAL) | n/p |
| 21 | - (50) | n/p | HM, NP | Cutaneous | No | Yes | *Rhizopus arrhizus* (Arm tissue) | Negative |
| 26 | - (50) | n/p | HSCT, NP | Sino-orbital | Yes | Yes | *Rhizopus arrhizus* (Orbital tissue) *Rhizopus arrhizus* (Sinus swab) | *Rhizopus spp.* |
| N/a* | - (50) | - (50) (BAL) | n/a | n/a | n/a | n/a | n/p | *Rhizopus spp.* |

Parallel testing was defined as serum sampling for Mucorales PCR within 7 days of another fungal test; *patient from outside U of U, **Demonstration of Mucorales in at least 2 unique body sites/systems (including histopathology), or evidence of disease in another body site/system consistent with mucormycosis with no other explanation or infectious process identified, ***If “angioinvasion” or “involving vessels” was noted by histopathology

**Supplemental Table 5. Adjudication and outcomes for University of Utah Mucorales positive patients**

| Pt # | Age | Sex | Host Factors | Histological evidence | Radiologic evidence; Clinical disease | PCR source | PCR (Ct)** | | Fungal culture, any time (source) | Pan-fungal sequencing | IMD?*** | Mucor-mycosis? **** | 30-day all-cause mortality | Treatment |
| --- | --- | --- | --- | --- | --- | --- | --- | --- | --- | --- | --- | --- | --- | --- |
| 1 | 22 | M | HM | Yes; "Morphologically consistent with mucormycosis" | Visual evidence; Cutaneous | *Tissue | + | 22.2 | **Rhizopus* spp. (skin tissue) | **Rhizopus* spp. | Proven | Proven | no | AMB, ISAV |
| 2 | 60 | F | HDCS | Yes; "Invasive fungal sinusitis" | Yes; Sinusitis | *Serum | + | 35.2 | *Candida tropicalis* (blood)  *Aspergillus fumigatus* (Lung tissue) | N/p | Proven | Possible | yes | n/a; deceased prior to results |
| 3 | 43 | M | HSCT | Yes; "Fungal elements observed" | Yes; Sinusitis | *Sinus tissue | + | 28.2 | **Rhizopus* spp. (sinus tissue) | **Rhizopus* spp. | Proven | Proven | no | AMB, ISAV |
| 4 | 19 | F | HM | Yes; "Morphologically consistent with mucormycosis" | Yes; Sinusitis | *Sinus Tissue | + | 19.7 | **Mucor* *circinelloides* (sinus tissue) | *Mucorales spp. | Proven | Proven | yes | AMB, POSA |
| 5 | 70 | M | HM | Yes; "Fungal forms present" | Yes; Pulmonary | *BAL | + | 31.9 | *Negative (BAL) | N/p | Proven | Possible | yes | AMB |
| 6 | 65 | M | SOT | Yes; "Morphologically consistent with mucormycosis" | Yes; Disseminated | *BAL  BAL  Serum | + | *26.7 30.2  34.2 | **Rhizopus* spp. (BAL)  *Rhizopus* spp. (Lung tissue, pericardial fluid, pericardial tissue, chest wall tissue) | N/p | Proven | Proven | yes | AMB, ISAV |
| 7 | 68 | M | HM, HSCT, NP | No | Yes; Pulmonary | *BAL | + | 39.2 | *Negative (BAL) | N/p | Possible | Possible | yes | AMB, POSA |
| 8 | 61 | M | HM, NP | Yes; "Fungal forms, consistent with Aspergillus" | Yes; Pulmonary | *BAL | + | 37.2 | *Negative (BAL) | N/p | Proven | Possible | yes | AMB, POSA |
| 9 | 60 | M | UDM | Yes; "Morphologically consistent with mucormycosis" | Yes; Sinusitis | *Tissue | + | 18.6 | **Rhizopus* *arrhizus* (sinus tissue) | **Rhizopus* spp. | Proven | Proven | no | AMB |
| 10 | 43 | M | UDM | Yes; "Morphologically consistent with mucormycosis" | Yes; Rhino-cerebral | *Sinus tissue | + | 25 | **Rhizopus* spp.  *Aspergillus flavus* (sinus tissue) | **Rhizopus* spp. | Proven | Proven | yes | AMB, POSA |
| 11 | 79 | F | HM | No | Yes; Pulmonary | *BAL | + | 20.9 | **Rhizopus* spp. (BAL) | N/p | Probable | Probable | yes | n/a; deceased prior to results |
| 12 | 15 | M | HDS | Yes; "Morphologically consistent with mucormycosis" | Yes; Pulmonary | *BAL | + | 31.3, 32.3 | **Rhizopus* spp. (BAL) | N/p | Proven | Proven | yes | AMB |
| 13 | 54 | M | HM | No | Yes; Pulmonary | *BAL | + | 35.1 | *Negative (BAL)  *Nakaseomyces* *glabratus* (Thoracentesis fluid) | N/p | Possible | Possible | yes | no |
| 14 | 61 | M | HDCS | Yes; "Morphologically consistent with mucormycosis with angioinvasion" | Yes; Sinusitis | Serum | - | 50 | *Rhizopus* spp. (sinus tissue) | N/p | Proven | Proven | no | AMB, ISAV |
| 15 | 61 | M | HM | Yes; "Morphologically consistent with mucormycosis" | Yes; Sinusitis | Serum | + | 39 | *Rhizopus* *arrhizus* (sinus tissue) | N/p | Proven | Proven | yes | AMB, ISAV |
| 16 | 30 | M | SOT | Yes; "Morphologically consistent with mucormycosis with angioinvasion" | Yes; Cutaneous | Serum | - | 45.5 | *Rhizopus* spp. (leg tissue) | *Rhizopus* spp. | Proven | Proven | no | AMB, POSA |
| 17 | 59 | F | HSCT, NP | N/p | Yes; Pulmonary | Serum | + | 35.8, 38.7 | Negative (BAL, CSF) | N/p | Possible | Possible | yes | AMB, ISAV |
| 18 | 48 | M | none | Yes; "Morphologically consistent with mucormycosis with angioinvasion" | Visual evidence; Cutaneous | Serum, BAL | - | 50, 50 | *Lichtheimia* *corymbifera* (leg tissue) | N/p | Proven | Proven | yes | AMB, POSA |
| 19 | 53 | M | UDM | Yes; "Morphologically consistent with mucormycosis" (sinus tissue) | Yes; Sinusitis | CSF | - | 46.9 | *Rhizopus* *arrhizus* (nasal tissue) | N/p | Probable | Probable | no | AMB, POSA |
| 20 | 31 | M | HM, NP | Yes; "Morphologically consistent with mucormycosis" | Yes; Rhino-cerebral | Tissue (fixed) | + | 26.8 | Negative (sinus tissue, BAL) | Negative | Proven | Probable | no | AMB, ISAV |
| 21 | 56 | F | HM, NP | Yes; "Morphologically consistent with mucormycosis with angioinvasion" | Visual evidence; Cutaneous | Serum | - | 50 | *Rhizopus* *arrhizus* (arm tissue) | Negative | Proven | Proven | no | AMB, ISAV |
| 22 | 79 | F | HM | No | Yes; Pulmonary | BAL | + | 20.9 | *Rhizopus* spp. (BAL) | N/p | Probable | Probable | yes | AMB |
| 23 | 5 wks | M | none | Yes; "Morphologically consistent with mucormycosis" | Visual evidence; Cutaneous | Serum | + | 36.7 | Negative (axilla tissue) | *Rhizopus* spp. | Proven | Proven | yes | AMB, POSA |
| 24 | 56 | M | SOT | Yes; "Morphologically consistent with mucormycosis" | Yes; Cutaneous | Serum | - | 48.2 | *Rhizopus* *arrhizus* (chest tissue) | N/p | Proven | Proven | no | AMB |
| 25 | 72 | F | HM | Yes; "Morphologically consistent with mucormycosis with angioinvasion" | Yes; Sinusitis | Serum | + | 39.1 | *Lichtheimia corymbifera* (sinus tissue) | N/p | Proven | Proven | Yes | AMB |
| 26 | 68 | M | HSCT, NP | Yes; "Morphologically consistent with mucormycosis with angioinvasion" | Yes; Sino-orbital | Serum | - | 50 | *Rhizopus arrhizus* (orbital tissue) | *Rhizopus* spp. | Proven | Proven | no | AMB, ISAV |
| 27 | 44 | F | UDM | Yes; "Fungal forms, consistent with Aspergillus" | Yes; Pulmonary | Serum | + | 39.4 | *Nakaseomyces glabratus* (BAL) | *Cunningham-ella* spp./ *Rhizopus* spp. | Proven | Proven | no | AMB, POSA |

*Indicates paired sample

**Ct<40.0 is considered positive

***See EORTC/MSG Criteria for IMD (Supplemental Table 1)

****See Mucormycosis Modified EORTC/MSG Criteria (Supplemental Table 1)

N/p: not performed, HM: hematologic malignancy; HSCT: hematopoietic stem cell transplant; UDM: uncontrolled diabetes mellitus; SOT: solid organ transplant; HDS: high dose corticosteroids; NP: neutropenia; IMD: Invasive mold disease; AMB: Amphotericin B; ISAV: Isavuconazole; POSA: Posaconazole

**Supplemental Table 6. Adjudication of potential false positive and negative Mucorales PCR results**

| **Pt #** | **Risk factors** | **Histopathology** | **PCR source (Ct)** | **30-day all-cause mortality** | **Treatment** | **Clinical Findings** | **Other Diagnostics** | **Final case assessment** |
| --- | --- | --- | --- | --- | --- | --- | --- | --- |
| **Potential False Positives:** | | | | | | | | |
| 2 | HDCS | Yes; Sinus tissue: "Invasive fungal sinusitis" | Serum (35.2) | yes | n/a; deceased prior to results | New lung lesions; Stroke/Brain infarct with multiple lesions concerning for septic/fungal emboli; | Also had candidemia and an *Aspergillus* positive culture from lung tissue | Sinus and/or lung lesions were compatible with mucormycosis |
| 5 | HM | Yes; Lung biopsy (RUL): "Fungal forms present" | BAL (31.9) | yes | AMB | New lung lesions | Mucor PCR resulted after patient was placed on comfort care/deceased. No further confirmatory workup available. | Lung lesions were compatible with mucormycosis |
| 7 | HM, HSCT, NP | N/p on lung tissue, only from brain | BAL (39.2) | yes | AMB, POSA | Ground glass opacities on chest CT; Also diagnosed with CNS toxoplasmosis | No other evidence of IMD or confirmatory testing | Treated for suspected pulmonary mucormycosis |
| 8 | HM, NP | Yes; Lung biopsy: "Fungal forms, consistent with *Aspergillus*" | BAL (37.2) | yes | AMB, POSA | History of *Aspergillus* pneumonia 6 months prior, the patient was on voriconazole at the time of biopsy; An acute/different process was suspected due to new lung cavitations | Patient was discharged to hospice/comfort care due to clinical status. No other confirmatory testing performed. | Treated for suspected pulmonary mucormycosis |
| 13 | HM | N/p | BAL (35.1) | yes | none | Necrotizing pneumonia, possibly due to aspiration | Clinical improvement without Mucorales treatment (i.e. received caspofungin). | Mucor PCR felt by clinical team to be falsely positive.  Not treated for pulmonary mucormycosis and improved |
| 17 | HSCT, NP | N/p | Serum (35.8, 38.7) | yes | AMB, ISAV | Worsening pulmonary disease with consolidations and cavitary lesions and neutropenic fever. Persistently positive serum Mucor PCR with no other positive laboratory findings. Unable to biopsy lesions due to patient status | Patient was discharged to hospice/comfort care due to clinical status. No other confirmatory testing performed. | Treated for suspected pulmonary mucormycosis |

| **Potential False Negatives:** | | | | | | | | |
| --- | --- | --- | --- | --- | --- | --- | --- | --- |
| 14 | HDCS | Yes; Sinus tissue:  "Morphologically consistent with mucormycosis with angioinvasion" | Serum (50.0) | no | AMB, ISAV | Sinusitis | Sinus culture growing *Rhizopus* spp. | Treated for proven sinus mucormycosis |
| 16 | SOT | Yes; Leg tissue:  "Morphologically consistent with mucormycosis with angioinvasion" | Serum (45.5) | no | AMB, POSA | Necrotic leg lesion | Leg tissue culture growing *Rhizopus* spp.  Pan-fungal sequencing from leg tissue positive for *Rhizopus* spp. DNA | Treated for proven cutaneous mucormycosis |
| 18 | None | Yes; Leg tissue: "Invasive fungal elements morphologically consistent with mucormycosis with angioinvasion" | Serum (50.0),  BAL  (50.0) | yes | AMB, POSA | Tissue ischemia following cardiovascular event with new necrotic leg lesion | Leg tissue culture growing *Lichtheimia corymbifera* | Treated for proven cutaneous mucormycosis |
| 19 | UDM | Yes; Sinus tissue: “Invasive fungal elements morphologically consistent with mucormycosis” | CSF (46.9) | no | AMB, POSA | Sinusitis | Sinus culture growing *Rhizopus* spp. | Treated for proven sinus mucormycosis |
| 21 | HM, NP | Yes; Skin biopsy: "Invasive fungal elements morphologically consistent with mucormycosis with angioinvasion" | Serum (50.0) | no | AMB, ISAV | Skin lesions and multiple new pulmonary nodules | Skin lesion biopsy culture growing *Rhizopus arrhizus (*formerly *R. oryzae)* | Treated for presumed disseminated mucormycosis |
| 24 | SOT | Yes; Chest wall incision site: "invasive fungal elements morphologically consistent with mucormycosis" | Serum (48.2) | no | AMB | Chest wall surgical site infection following lung transplant | Wound culture from chest wall surgical site growing *Rhizopus arrhizus (*formerly *R. oryzae)* | Treated for proven cutaneous mucormycosis |
| 26 | HSCT, NP | Yes; Sinus tissue: "invasive fungal elements morphologically consistent with mucormycosis" | Serum (50.0) | no | AMB, ISAV | Invasive sinusitis with extension to the orbit | Sinus tissue culture growing *Rhizopus arrhizus (*formerly *R. oryzae)* | Treated for proven rhino-orbital mucormycosis |

*No Mucorales identified by culture

N/p: not performed, HM: hematologic malignancy; HSCT: hematopoietic stem cell transplant; HDCS: high dose corticosteroids; NP: neutropenia; IMD: Invasive mold disease; AMB: Amphotericin B; ISAV: Isavuconazole; POSA: Posaconazole
